# Conceptualising social accountability as an attribute of medical education

**DOI:** 10.4102/phcfm.v12i1.2213

**Published:** 2020-02-18

**Authors:** Amy Clithero-Eridon, Danielle Albright, Andrew Ross

**Affiliations:** 1 Department of Family and Community Medicine, University of New Mexico School of Medicine, Albuquerque, New Mexico, United States; 2 Department of Emergency Medicine, University of New Mexico School of Medicine, Albuquerque, New Mexico, United States; 3 Department of Family Medicine, University of KwaZulu-Natal, Westville, Durban, South Africa

**Keywords:** social accountability, medical education, professional identity formation, South Africa

## Abstract

**Background:**

Health professionals need to be both person- and community oriented to improve population health. For educators to create socially accountable physicians, they must move learners from understanding social accountability as an expectation to embracing and incorporating it as an aspect of professional identity that informs medical practice.

**Aim:**

The aim of this article was to assess the degree to which medical students, preceptors and community mentors understand the concept of social accountability.

**Setting:**

The setting is the KwaZulu-Natal Province in Durban, South Africa.

**Methods:**

Using an observational design, we surveyed 332 participants, including the first- and sixth-year medical students, physician preceptors and community mentors.

**Results:**

Whilst most respondents understood social accountability as requiring an action or set of actions, it was defined by some as simply the awareness one must have about the needs of their patients, community or society at large. Some respondents defined social accountability as multi-dimensional, but these definitions were the exception, not the rule. Finally, most respondents did not identify to whom the accountable party should answer.

**Conclusion:**

Whilst the development of professional identity is seen as a process of ‘becoming’, the ability to define and understand what it means to be socially accountable is not a linear process. Assessment of this progress may start with comprehending how social accountability is understood by students when they begin their education and when they are graduating, as well as in knowing how their educators, both clinical and community, define it.

## Introduction

A core competency at the Nelson R. Mandela School of Medicine (NRMSM), located in Durban, KwaZulu-Natal Province, South Africa, is for medical students to become physicians who are change agents and committed to their communities.^1^ Some health professional educators contend that their efforts need to be both person- and community oriented to improve population health.^2^ This includes not only the technical expertise to care for individuals but also a service orientation and an ethical commitment to the communities in which they practice. A socially accountable physician is one who has a deep and profound understanding of his or her community responsibilities from having been trained in the community with a view towards population health and eliminating health inequities in partnership with community members.^3^ In order for health profession educators to create socially accountable physicians, they must move learners from the awareness of accountability as an expectation to action by incorporating it as an aspect of professional identity that informs medical practice.

Whilst social accountability in health professional education is ‘gaining traction internationally as a mechanism for combatting health inequities and advancing universal health coverage’,^4^ it is not a universally understood concept.^5,6,7^ In 1995, the World Health Organization (WHO) defined social accountability of medical schools as:

[*T*]he obligation of medical schools to direct their education, research and service activities towards addressing the priority health concerns of the community, region, and/or nation they have the mandate to serve. The priority health concerns are to be identified jointly by governments, healthcare organizations, health professionals, and the public. (p. 3)^3^

Medical education leaders from around the world adopted the Global Consensus on Social Accountability of Medical Schools.^8,9^ This consensus defined the gradients of social obligation. The obligation begins with social responsibility or the recognition that issues within the community exist. The next step within the progression is social responsiveness, whereby the organisation acts on the issues. Social accountability is the culmination with a demonstration of impacts such as producing graduates who will practice within the community where they have been taught and are able to address the issues effectively.

This definition has served as the basis for defining social accountability amongst most educational institutions. As a result, research on social accountability has mostly focused on conceptualising indicators for curricula and programmes that aim to incorporate the common principles of social accountability. These principles include, but are not limited to, recruitment of students that match the community profile, strong emphasis on primary care and learning within communities, and graduates practising in underserved communities.^4,10^

There is a need for further exploration of defining what the term means from the perspective of those for whom the expectations of accountability are being set and how these definitions change as they progress from learner to community physician. Whilst the development of professional identity is seen as a process of ‘becoming,’^11,12^ the ability to define and understand what it means to be socially accountable is not a linear process. The acquisition of social awareness and technical skills (informing) and the creation of a professional identity (forming) are co-occurring, which ultimately yields leaders who are committed to improving population health.^2^ Assessment of this progress may start with comprehending how social accountability is understood by students when they begin their education and when they are graduating, as well as knowing how their educators, both clinical and community, define it. We address a gap in the literature by exploring written definitions for social accountability from multiple perspectives, including the first- and final-year medical students, community medical student preceptors and community leaders who mentor first-year students.

## Methods

### Design

We surveyed 332 participants from both NRMSM and KwaZulu-Natal Province. This study is an observational design.

### Sample

The sample consists of four groups. The first group composed of all first-year medical students (*N* = 246) who enrolled in a professional development course at NRMSM^13^ and who completed an online questionnaire as part of their final course evaluation. The second group composed of a convenience sample of sixth-year NRMSM medical students (*N* = 10) who completed a paper survey after a 7-week family medicine rotation. The third group composed of physician preceptors who completed paper-based surveys following the supervision of sixth-year medical students as part of their practice at district hospitals within the KwaZulu-Natal Province (*N* = 41). They were recruited by the researcher A.R. based on personal affiliation. The fourth group in the sample are community mentors supervising the first-year medical students as part of the professional development course identified above (*N* = 47). They completed a paper-based survey. In total, 303 surveys were distributed to both student groups and community mentors. From these groups, we received 291 completed surveys, resulting in a response rate of 96%. The number of surveys distributed to physician preceptors is unknown, and therefore a full response rate cannot be calculated.

### Data collection

We constructed and distributed surveys to NRMSM medical students, physician preceptors and community mentors between April and June 2017. The surveys are part of a broader exploration of NRMSM’s progress towards social accountability. These surveys were four separate surveys. Results from some of these surveys are reported elsewhere or are currently in review.^14^ Each survey, regardless of version, included an open-ended question asking respondents to define the term social accountability, specifically, ‘What does the term “social accountability” mean to you?’ We pulled responses to this question from all four surveys for analysis. Responses were emailed to researchers at the University of New Mexico School of Medicine in Albuquerque, NM, USA.^15^ We transcribed responses to the question of interest verbatim into Microsoft Excel.

### Data analysis

We used a qualitative analytic approach. The data were inductively coded by two independent researchers (A.C.E. and D.A.), who used a consensus process to identify categories and primary themes in respondent understandings of social accountability. We next approached coding with four analytic questions, including the identification of (1) awareness versus action orientation, (2) for what one is to be held accountable, (3) who is to be held accountable and (4) to whom one is answerable. Frequencies are reported to give context to these findings.

### Ethical consideration

The Biomedical Research and Ethics Committee of the University of KwaZulu-Natal approved the study design on 13 March 2017 (Protocol reference number: HSS/0119/017D). Each participant provided written informed consent prior to participation in the study.

## Results

Of the 332 survey respondents, 21 did not provide a response to the question to define social accountability. We report percentages based on the 311 cases with definitions in the following sections. Specifically, 238 responses from the first year medical students, resulting in a 97% question response rate; 8 responses from sixth-year medical students (80% question response rate); 32 responses from physician preceptors (78% question response rate); and 33 responses from community mentors (92% question response rate). The request for demographic information about respondents varied depending on the survey version:

- No demographic information was collected on first-year students.
- The age range of the sixth-year students was 20–30 years, with 78% under age 25.
- The physician preceptors represented a range of specialties. Respondents were predominantly Family Medicine/General Practice physicians (63%), working in a district health facility (95%) and an urban setting (71%) in a population greater than 100 000 persons.
- Amongst community mentors, 71% were over the age of 35. Approximately 75% were managers, staff or volunteers at the community organisation. The remainder were nurses or social workers. Ninety per cent of community respondents indicated their population was considered disadvantaged.

Table 1 documents the number of respondents returning a survey with a definition of social accountability.

**TABLE 1:** Number of respondents providing a definition of social accountability.

Person type	Number of returned surveys	Number of respondents providing definition
First-year medical students	246	238
Sixth-year medical students	10	8
Physician preceptors	41	32
Community mentors	47	33

**Total**	**332**	**311**

*Social accountability is defined by some as simply the awareness one must have about the needs of their patients, community or society at large.* Awareness is simply the acknowledgement of the expectation that providers have a responsibility to their patients as both individuals and members of the community and society at large. Twenty-one per cent of respondents provided definitions that fall into this category. One way respondents defined social accountability was as an awareness of the responsibility to know about community concerns or to be ‘mindful’ of social needs or priorities. ‘According to my understanding, social accountability refers to your responsibility as an individual to being mindful of the emerging social concerns and priorities of the community you serve’ (First year). Another wrote, ‘social accountability is the measure of one’s mindfulness and commitment to serving the society’ (First year).

Awareness was largely described as a personal characteristic, an intrinsic understanding of one’s role as a citizen or health care provider. A preceptor defined social accountability as an ‘attitude of servitude toward the community within which you work and reside’. Similarly, a sixth-year student wrote that it is ‘taking one’s social responsibilities to heart’.

Physician preceptors were more specific about the types of social characteristics one should be aware of and how it may impact the patient care experience. One clinician described awareness as having an impact on the patient’s care experience by stating, social accountability is ‘taking the patients’ social and economic circumstances into account when treating them in terms of follow up and cost and management’. Another wrote that social accountability was ‘understanding the health and social repercussions of decisions made as a doctor, in the treatment, management and interaction with patients’ (Physician preceptor).

Whilst these respondent definitions acknowledged that there is an issue and something needs to be done, they did not include a direct action component to address either individual social circumstances or community concerns. Table 2 shows the distribution of responses by awareness or action orientation and person type.

**TABLE 2:** Primary theme of social accountability definition by respondent type.

Theme	First-year medical students (*N* = 238)		Sixth-year medical students (*N* = 8)		Physician preceptors(*N* = 32)		Community mentors(*N* = 33)		Total (*N* = 311)	
	*N*	%	*N*	%	*N*	%	*N*	%	*N*	%
Awareness	49	21	4	50	6	19	5	15	64	21
Action	189	79	4	50	26	81	28	85	247	79

**FIGURE 1:**
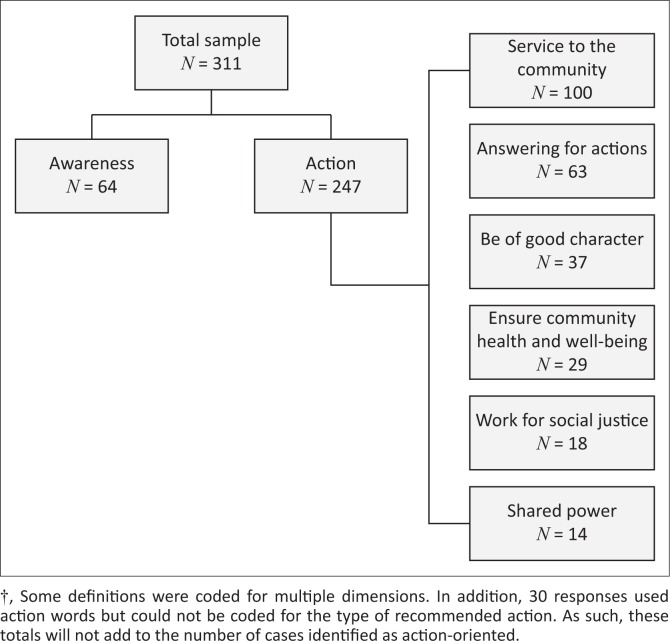
Distribution of primary dimensions of action-oriented definitions of social accountability.?

*Most respondents understood social accountability as requiring an action or set of actions*. These definitions included expressed beliefs that action is required to address the individual’s needs, take into account actions consistent with the socio-economic circumstances of the individual or attend to the health of the community more broadly. Seventy-nine per cent of respondents provided definitions that fall into this category. We identified six categories of actions in respondent definitions of social accountability, presented here in order of frequency from highest to lowest: being of service to the community, answering for one’s actions, being of good individual character, ensuring the health and well-being of the community or society as a whole, working for equality or social justice and shared power between institutions and the public. Figure 1 shows the distribution of responses for each action-oriented dimension by respondent type.

*Be of service to the community.* Respondents most commonly defined social accountability as inclusive of the idea that one should be of service to the community or society at large. This dimension appeared in 40% of action-oriented definitions. Respondents commonly suggested that social accountability ‘means to give back to your community’. Being of service included being available or present. A first-year medical student wrote, ‘it means to easily avail yourself to other people to help them’. Some suggested that individuals should be more than present and get involved in institutional activities, by ‘being socially responsible in terms of civic engagement or other community services’ (First year). Others identified not only service to the community but anticipated outcomes. A community member wrote:

‘[*E*]ach community member has a role to play in responsibility in uplifting their community with their unique strengths and striving towards a common goal of creating and maintaining a safe, healthy, and happy community.’

*Answering for one’s actions.* The second most common dimension in respondent definitions of social accountability was answering for one’s actions. This dimension appeared in 26% of definitions. For some, this meant being prepared or able to ‘explain your actions’ (First year). Others were more specific about the site of action. Another first-year student wrote that social accountability was ‘being responsible for your actions in the social setting and taking responsibility for whatever goes wrong’. Answering for one’s actions was also extended to organisations, with one defining social accountability as ‘the ability of an organization to account for acts’ (First year). Many framed actions taken by physicians and health care organisations as having consequences for both patients and the community as a whole.

*Being of good individual character*. Some respondents defined social accountability in terms of one’s individual character. Fifteen per cent of definitions included some form of call to be ‘honest,’ treat people with ‘respect and dignity’, or to be ‘transparent’. One first-year student wrote: ‘being able to answer for what you do even if it was wrong. Standing for telling the truth and nothing but the truth even when you may get into a lot of trouble.’ The responsibility for transparency was identified as part of medical practice, but sometimes placed on organisations and institutions rather than individuals:

‘To me social accountability means more transparency such as in public serving, for example, government should not do things without consulting the community about how do they feel and their views based on the project to be done because if it happens that the community is affected, then such organization should be accounted for the consequences of their doing.’ (First year)

*Ensuring the health and well-being of the community*. The fourth category of action-oriented definitions was a call for ensuring positive health and well-being outcomes for patients, members of the community and society as a whole. This dimension was present in 12% of respondent definitions. For some, this focused specifically on the health care provider:

‘My understanding of social accountability is that healthcare professionals are responsible for the healthcare of the nation and so must use their knowledge and skills to address public healthcare issues in order to improve and benefit the lives of the population in the community that they are based in.’ (First year)

Others focused specifically on health and wellness education. A physician preceptor wrote that social accountability is ‘being accountable to the community in your practice. To do more than just treat with medications, but to educate the community about appropriate health practices.’ Others called for treating the community ‘holistically’ and working towards a healthy ‘environment’.

*Working for social justice*. Another way respondents defined social accountability was in terms of working for equality and social justice. Seven per cent of definitions included a social justice dimension. These definitions included a call to be aware of and teach others about their rights, working to improve the lives of the disadvantaged and eradicating social problems that create disadvantage. Most focused on knowing your rights, promoting the rights of others and holding the government responsible for ensuring people’s rights are respected:

‘Social accountability is taking responsibility as a citizen and being aware of the influence that I have on the people and communities around me. Through this awareness is knowing my rights as a citizen and demanding for my rights to be acknowledged by the government. It is also reminding the government of the responsibilities they have to the citizens of this country.’ (First year)

*Shared power*. The final and least common element of respondent definitions for social accountability was the requirement of shared power between institutions and the public. Six per cent of definitions pointed to sharing power and included themes of involving the community in assessing and evaluating health care and exacting accountability of organisations and the government through public decision-making processes. A preceptor defined social accountability as ‘being accountable to community to involve them in assessments of health needs and proposed interventions’. Another respondent described the need for collaboration to ensure that organisations and government are meeting their responsibilities, ‘social accountability is the involvement of individuals, communities or organizations in holding service providers or government accountable for issues and making them responsive to the needs of citizens’ (First year).

*Some respondents defined social accountability as multi-dimensional, but these definitions were the exception not the rule*. Just under 19% of action-oriented definitions were coded for multiple dimensions. Most of these were a single dimension paired with being of service to the community. For example, a preceptor defined social accountability as both answering for one’s actions and service to community: ‘to be accountable to society in a way which displays expertise in my field while engaging individually with the socio-cultural needs and interests of the community’. Another respondent combined being of good moral character with a social justice call for non-discriminatory treatment of patients, by defining social accountability as ‘having respect for every person, treating every patient the rich or the poor and being honest to your job as a doctor’ (First year).

Social accountability definitions also differed amongst respondents on the questions: who is responsible and to whom the responsible party is answerable to for this action.

*The majority of respondents identified the individual as the entity to be held socially accountable.* Respondents across all person types for both awareness (61%) and action-oriented (83%) definitions identified the ‘individual’ or ‘self’ as the responsible party for social accountability. A first-year medical student described accountability as ‘when a person is aware of the commitments of the community surroundings and its concerns about human rights, healthy, privacy and betterment of others’. Another provided an action-oriented definition, but still identified the individual as responsible:

‘Social accountability means that you’re held responsible for any action that you take regarding your social life and other aspects that it might include as a whole. The decisions that you might take can either be negative or positive, but whatever consequence that will come out it’ll be in your hands to take responsible actions of it.’ (First year)

Fewer respondents identified at least one external entity to whom one would or should have to answer to with regard to the enforcement of social accountability. Respondents identified organisations as the second most common responsible party in both awareness and action-oriented definitions. The third most common was collaborative responsibility (e.g. public/organisation or individual/organisation). For example, a first-year medical student implicated both organisations and individuals by writing, ‘it is about making academic institutions, health systems and health professionals accountable for results’. This was followed in frequency by the identification of the public and government as responsible parties, respectively.

**FIGURE 2:**
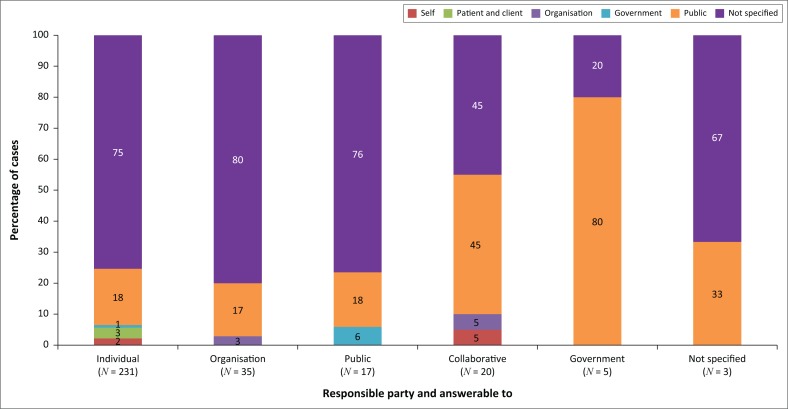
Identification of who is the responsible party and to whom the responsible party must answer by primary dimension.

*Most respondents did not identify to whom the accountable party should answer*. Just under 75% of responses did not specify to whom the responsible party answers. The public or community was the most common entity identified (*N* = 65, 21%). The remaining respondents identified the patient or client, self, government or organisations as the party that enforces social accountability. Figure 2 shows the distribution of dimensions for the responsible party and to whom the party must answer.

*Defining social accountability is not an easy task*. Respondents often used forms of the words ‘responsible’ and ‘accountable’ in their definitions. Just under 42% of respondent definitions included a form of the term ‘responsible’. For some, the definition did not include an identified object of responsibility, ‘being socially responsible’ (First year). For others, the object was identified. A sixth-year student wrote: ‘social accountability is being responsible for your behavior in social situations’. Similarly, a preceptor defined social accountability as ‘responsible for your actions’. Objects of responsibility also included other awareness and action-oriented dimensions.

Many of the written definitions for social accountability included the use of the words ‘accountable’ or ‘accountability’. In fact, 22% of respondents used at least one of these words as part of their definition. Less than 2% used a form of the term ‘responsive’. A first-year medical student wrote: ‘social accountability is the involvement of individuals, communities or organizations in holding service providers or government accountable for issues and making them responsive to the needs of citizens’. In all but one of these definitions, respondents described making an external entity responsive to community needs or well-being. A preceptor wrote: ‘Social accountability means being responsible for and responsive to the challenges of one’s surrounding society, regardless of whether one experiences those challenges personally.’ Although this response places the onus on the individual, there is an acknowledgement that accountability is an external and not an individual’s characteristic.

## Discussion

This observational study was undertaken to describe how medical students, physician preceptors and community mentors understand social accountability. We also aimed to explore the complexity of social accountability from these different points of view to assess the possible evolution of definitions across the professional development spectrum. We found that most of the respondents in our sample proscribed an action orientation to social accountability. We identified six types of recommended action: being of service to the community, answering for one’s actions, being of good individual character, ensuring the health and well-being of the community or society as a whole, working for equality or social justice, and shared power between institutions and the public.

Professional identity formation occurs by both formal and informal education as well as explicit and implicit exposures.^11,12^ One study in a South African medical school found that most final-year students believed social accountability was already built into a person’s values and belief systems.^16^ This appears to be true for our respondents, who largely defined social accountability as a responsibility to serve the community. Our results are primarily driven by the responses of first-year medical students. Other studies have found that students often lose their altruism as they progress in their education because of the hidden curriculum effect.^17^ Some medical students identified that social accountability was subconsciously realised through their educational progression, albeit something that was a future not a current obligation.^18^ Yet, studies have shown that medical students with a clear perception of social mission with opportunities to directly experience it during their education have a better understanding of not just the term, but they operationalise it within their own practice.^19,20^ Social awareness and a proclivity towards social justice require the development of a socially responsible value and belief system. However, it is not sufficient for creating socially accountable medicine in the absence of institutional responsibility. This suggests the need for more cohort-based and longitudinal research to assess both uptake and persistence of social accountability concepts across medical education and entry into the workforce.

One of the characteristics of social accountability is fostering partnerships between health care organisations and the communities they serve. Whilst most respondents did not identify to whom the responsible party must answer, those that did situated the community or the public at large as the entity to which social accountability is owed. We have included community mentors who we believe are not only instructors but also role models for the students in their professional development. Further, they are an integral part of the social institutions that shape the provision of health care, and thus, it is essential to include them when assessing the definition of social accountability. Prior research has shown that community members can add a rich dimension to the definition, including caring for one another.^21^ Relatively few studies have considered mentorship by non-physicians in the health care environment. To properly develop social accountability, community mentors must have a voice and role in health professional education beyond serving as project and clinical sites.

The literature on social accountability is largely situated in the meso-level of social organisation (community, organisations and governments), not the individual.^4,8,9,22^ The WHO definition of social accountability firmly places accountability as an institutional responsiblity.^4^ We observed that most respondents situated the individual as the responsible party and did not identify to whom these individuals should have to answer. Few attributed responsibility to the organisation or institution. This suggests that what may be lacking is the recognition of the institution’s role in serving the community, apart from the responsibilities of individuals. This same gap may also explain why we did not see many respondents including shared power in their definition of social accountability. Whilst our work is not comparative in nature, these findings do suggest a need to place future studies of social accountability in the cultural and institutional context of South Africa’s health care systems and explore how strengths and weaknesses of these institutions may facilitate or inhibit social accountability.

## Limitations

This work was a retrospective analysis of responses to an open-ended survey question, which had several limitations. The uneven distribution of respondents by person type interfered with our ability to compare differences between groups. This resulted in an underrepresentation of sixth-year medical student responses. We also had to rely on the existing questionnaire. The survey methodology did not allow for probing or follow-up on components of the definition. We tried to account for various dimensions by coding who and what, if presented. However, survey respondents were not instructed to address these dimensions. Many responses were not complete enough to demonstrate an understanding of social accountability at any of the gradients of social obligation described by Boelen and colleagues.^23^

We set out to get a better sense of whether student definitions of social accountability differed between first-year and sixth-year students. A socially accountable physician is a community-engaged person and a central component to a healthy population.^24^ The Independent Global Commission on Education for Health Professionals classified three levels of learning to achieve the vision of transformative education: (1) *Informing: acquiring skills, (2) Forming: creating professional identity and (3) Transforming: creating leaders who can effectively lead health systems and improve population health*.^2^ Whilst there were some observable differences in the attribution of action between the two student groups, ultimately, the limited sample size in the sixth-year group prevents us from concluding a real difference exists. Further, we were not able to adequately explore the developmental understanding of social accountability in this study. A more comparable sample or longitudinal work with a group of students as they matriculate through their medical education is needed to shed light on both the dimensions of social accountability and the processes of ‘informing’ and ‘forming’ across the course of career development.

## Conclusion

With the increasing global focus on producing socially accountable health care systems, it is imperative that medical schools not only design accountable programmes and structures but also find a way to evaluate the impact of these interventions on the student population and eventual workforce. This project serves as the first step in assessing the degree to which medical students, preceptors and community mentors define the concept of social accountability. Our findings suggest that the action orientation of social accountability was commonly understood, but the organisational and institutional context in which it is embedded was not. We recommend future research probe for the various dimensions of social accountability with a cohort of medical students and practising physicians over time.
